# The mechanics of masterpieces

**DOI:** 10.1038/s44172-023-00060-9

**Published:** 2023-03-03

**Keywords:** Mechanical engineering

## Abstract

Dr. Emanuela Bosco describes her research into mechanical processes which affect the stability and longevity of historical artifacts.

**Figure:**
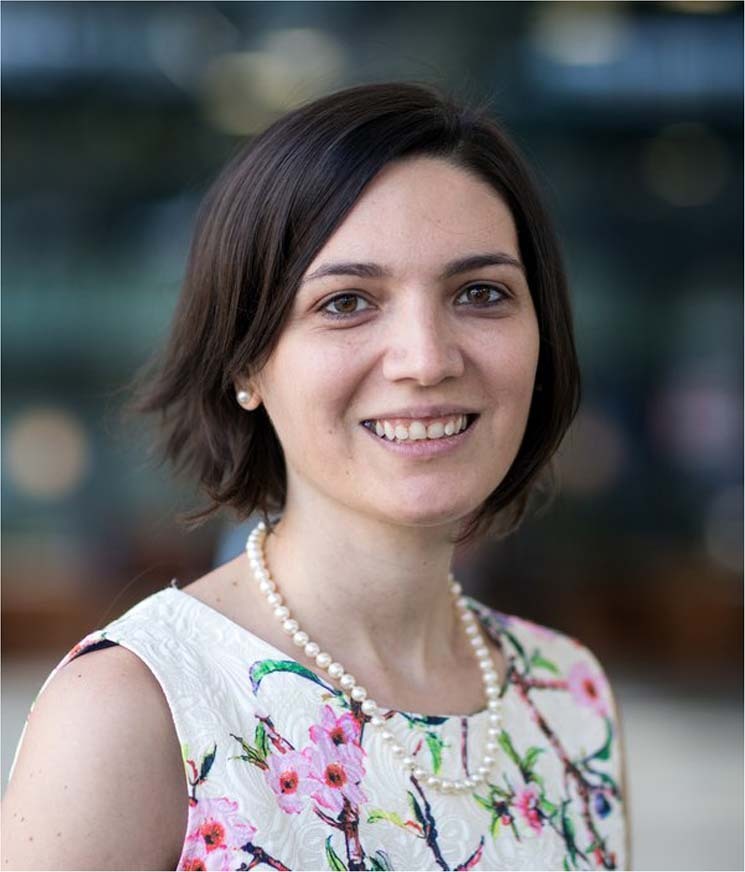
Angeline Swinkels

*Emanuela Bosco* is an Associate Professor in the Chair of Applied Mechanics and Design at Eindhoven University of Technology. Her research focuses on computational mechanics, in particular, on multiscale methods for understanding degradation processes in a variety of different materials and applications. We asked Dr. Bosco to tell us about her research into the degradation of historical artifacts and how she works with museum conservators to create strategies for preservation.

What originally got you interested in computational mechanics?

During my studies in structural engineering, I was fascinated by the versatility and the broad field of application of (computational) mechanics. Using mechanics, we can describe and simulate a wide range of real-world physical phenomena relevant to various engineering and technological problems. Moreover, I find it amazing that the governing equations of very different processes in nature have strong analogies. For example, I could not imagine two applications more diverse than the chemical degradation of historical paintings and the biochemical corrosion of cement in sewerages. Yet, the mathematical structure of the chemo-mechanical formulation that describes the two degradation processes is very similar!

What would you be if you weren’t a scientist and why?

A musician, a violist. I have studied as a classical musician at the Conservatory of my hometown, Brescia, in Italy. Until I completed my Ph.D., I also worked professionally in several Italian orchestras. After I moved to the Netherlands, I had to choose my career path. I decided to pursue academia instead of music. That was a hard decision! I am still playing and am occasionally involved in classical concerts in Eindhoven. I love music and art in general. This is one of the reasons why I find it so exciting to apply my scientific background to study the degradation of cultural heritage materials.

Part of your work involves studying the degradation of historical art. What sorts of questions are you investigating?

My goal is to advance the fundamental understanding of material degradation in various classes of cultural heritage materials. Degradation is often due to the interaction between mechanical and chemical processes and the variation of environmental conditions. I develop integrated modeling and experimental strategies to unravel the origins of observed degradation.

I study different degradation mechanisms that affect the appearance and integrity of various art objects—mostly oil paintings, wooden artifacts, and historical paper objects. The insights gained from my research, combined with the practical experience and knowledge of conservators, can lead to interventions to prevent or delay the degradation mechanisms.

One relevant problem for paint conservation is to better understand how relative humidity and temperature variations affect cracking in the paintings. The most common cracking mechanism is crack channeling, usually termed “craquelure.” A channeling crack may further deflect at the interface between the paint and the support, generating interfacial delamination that can ultimately lead to flaking and loss of the paint. This is the most dangerous failure scenario. In conservation practice, paintings with craquelure are considered to hold historic and aesthetical value (“patina”). Therefore, craquelure is not regarded as critical, provided delamination is absent. On the contrary, channeling cracks with delamination present should be a serious concern and require immediate precautionary measures before paint loss occurs. For example, “A wheatfield with cypresses” by Vincent van Gogh was at risk of paint loss due to delamination, requiring urgent conservation treatments. To limit cracking processes, the current practice is to maintain very restrictive climate conditions in museums. However, this involves huge costs and unsustainable energy policies. My research aims to predict which type of failure scenario will occur under which humidity and temperature variation ranges. A possibly broader environmental fluctuation can be suggested to conservators, enabling more efficient energy use while guaranteeing a safe condition for the art object. The sustainability point is crucial, especially nowadays!

Besides paintings, I am interested in wooden artifacts, particularly their aging response and the effect of moisture variation on the development of mechanical damage. My research group and collaborators analyze oak samples of microscopic dimensions dating from different historical periods—from 1300 AD onwards. These specimens were provided by the Rijksmuseum, which has a collection of old (but not artistic) material which can be used for experimentation. First, with computer tomography, we analyze the morphology of the specimens. Then we mechanically test the samples and measure their sensitivity to moisture. We compare these tests with a model that predicts how the material will respond over time under the influence of moisture and temperature. This information is relevant for conservators in defining sustainable energy policies.

Finally, I work on the acid-catalyzed chemo-mechanical degradation of historical documents and paper objects in museums, libraries, and collections. For this purpose, my research group and I developed a model that simulates the chemo-mechanical degradation process as a function of the environmental specifications and acidity of paper. From the simulation results, we constructed isochrone degradation maps, which enable a straightforward estimation of the expected lifetime of paper artifacts under specific acidity, temperature, and relative humidity conditions. This is relevant for conservators since it suggests the appropriate climate ranges for which historic paper degradation can be mitigated or delayed.

Tell us more about the exciting pieces you have worked on and their unique challenges.

I have had the privilege to provide a small contribution to “Operation Night Watch”. Since 2019, Rijksmuseum (Amsterdam, the Netherlands) has been conducting an extensive research project on “The Night Watch” - the most renowned masterpiece of Rembrandt. The project aims to conserve the painting for the future in the best way possible. Together with my research group and collaborators, we developed a methodology to characterize the mechanical response of individual natural fibers of microscopic dimensions. We applied the method to a set of original fibers and fiber threads extracted from the canvas of The Night Watch. The performed tests revealed that the original canvas is in an advanced degradation state, and the structural load of the painting is carried by the (more recent) lining canvas.

In another project, I studied the formation of metal soaps, a crucial degradation phenomenon afflicting many oil paintings in museum collections worldwide. Metal soaps form due to a chemical reaction between the oil paint and the metal ions from the pigments. They appear as opalescent hazes on the paint surface or large protrusions visible to the naked eye, deforming the paint layers. Additionally, metal soap formation introduces stresses in the paint, which can initiate mechanical damage. This phenomenon was observed in masterpieces of Rembrandt (for instance, in the Portraits of Marten Soolmans and Oopjen Coppit, jointly owned by the Rijksmuseum and the Louvre) and Johannes Vermeer (for example, View of Delft, located at the Maurithuis in The Hague). In this research, I closely interacted with conservators and conservation scientists working on these paintings and had the opportunity to see them out of museums and almost touch them, which was terrific.

What are the challenges for engineers and scientists working together with historians and museum curators working across disciplines? And how have you solved those challenges?

One of the greatest challenges of this highly multidisciplinary research is to establish a common understanding between experts in different disciplines. To solve this challenge, it is important to define clear communication between the involved researchers. For example, I recently participated in a large EU Horizon 2020 project, CollectionCare, which involved academics (engineers, physicists, conservator scientists), companies, museum curators, and cultural heritage professionals. At the beginning of the project, we carried out a thorough analysis of the project’s needs in the different areas, and we prepared a document establishing a unified use of terminology for all the consortium partners. In this sense, it is also essential to trust and rely on each other’s expertise and to be humbly open to learning in areas that do not cover your field of research. Another challenge is communicating and disseminating results and knowledge generated by the research to reach a larger community of conservators and experts in the cultural sphere. For this, I find it very useful to organize workshops open to a broader public to share my research outcomes. The interactions during these events also enable me to identify critical open issues in the conservation community, which helps me define future research. For instance, inspired by these interactions, my research group and I are currently investigating the occurrence of moisture-induced wrinkling and buckling in historical paper documents. This well-known problem still needs to be studied and better understood.

Finally, if you could work in one museum, one piece of artwork, or one historical artifact, what would it be and why?

The British museum! I find it one of the most impressive museums in the world. Its collection includes art pieces made of materials I never studied, for instance, stone, marble, etc. It would be a great challenge for me to learn and gain insight into the critical degradation phenomena of these materials.

*This interview was conducted by Rosamund Daw, Chief Editor, Communications Engineering.*

